# Norovirus Recombination in ORF1/ORF2 Overlap

**DOI:** 10.3201/eid1107.041273

**Published:** 2005-07

**Authors:** Rowena A. Bull, Grant S. Hansman, Leighton E. Clancy, Mark M. Tanaka, William D. Rawlinson, Peter A. White

**Affiliations:** * University of New South Wales, Sydney, New South Wales, Australia;; † University of Tokyo, Tokyo, Japan;; ‡ Prince of Wales Hospital, Randwick, New South Wales, Australia

**Keywords:** gastroenteritis, Recombinant norovirus, phylogenetic analysis, breakpoint, reverse transcriptase polymerase chain reaction, subgenomic RNA

## Abstract

Norovirus (NoV) genogroups I and II (GI and GII) are now recognized as the predominant worldwide cause of outbreaks of acute gastroenteritis in humans. Three recombinant NoV GII isolates were identified and characterized, 2 of which are unrelated to any previously published recombinant NoV. Using data from the current study, published sequences, database searches, and molecular techniques, we identified 23 recombinant NoV GII and 1 recombinant NoV GI isolates. Analysis of the genetic relationships among the recombinant NoV GII isolates identified 9 independent recombinant sequences; the other 14 strains were close relatives. Two of the 9 independent recombinant NoV were closely related to other recombinants only in the polymerase region, and in a similar fashion 1 recombinant NoV was closely related to another only in the capsid region. Breakpoint analysis of recombinant NoV showed that recombination occurred in the open reading frame (ORF)1/ORF2 overlap. We provide evidence to support the theory of the role of subgenomic RNA promoters as recombination hotspots and describe a simple mechanism of how recombination might occur in NoV.

Noroviruses (NoV) are divided into 5 genogroups (I–V) based on genome sequence (1). NoV genogroups I and II (GI and GII) are now recognized as the predominant worldwide cause of outbreaks of acute gastroenteritis in humans (2,3). NoV are small round virions 27–35 nm in diameter and possess a single-stranded, positive-sense RNA genome of 7.5 to 7.7 kb. The genome includes 3 overlapping open reading frames (ORFs) (4). The first ORF (ORF1) encodes a polypeptide with regions of similarity to helicase, cysteine proteinase, and RNA-dependent RNA polymerase (RdRp)-encoding regions of picornaviruses (5). ORF2 encodes a viral capsid protein (VP1), and ORF3 encodes a minor structural protein (VP2) associated with VP1 stability (6).

RNA recombination is among the major driving forces of viral evolution (reviewed in [7,8]). Recombination in viruses can greatly affect phylogenetic groupings, confuse molecular epidemiologic studies, and have major implications in viral vaccine design. A recombinant NoV can be defined as one that clusters with 2 distinct groups of NoV strains when 2 different regions (normally the capsid and polymerase) of the genome are subjected to phylogenetic analysis. The prototype Snow Mountain virus was the first reported naturally occurring recombinant NoV (9). Recently, 4 additional naturally occurring human recombinant strains have been reported: Japanese isolates Saitama U1 and the only reported GI recombinant WUG1 (10), the Thai isolate Mc37 (11), and Arg320 from Argentina (12) (Table). One recombinant strain closely related to Saitama U1 and 2 strains closely related to Mc37 have also recently been reported (13). Furthermore, outside of NoV GII but within the *Caliciviridae*, 2 recombinant NoV genogroup III strains associated with diarrhea in cattle (14,15) and a recombinant sapovirus (16) have also recently been reported. Analysis of these recombinants has suggested that the recombination points (or breakpoints) were near the ORF1/ORF2 overlap (9–12,14–16); however, this hypothesis has not been proven.

**Table:** Norovirus (NoV) recombinant strains and their close relatives

Prototype NoV recombinant strain (ref.)*	Sequence length		Parental strain‡		Genotype of recombinant§			Related strains (>96%)	
	RdRp†	Capsid	RdRp†	Capsid	RdRp†	Capsid	Breakpoint¶	Isolate name	Accession no. (ref.)
Arg320/1995/AR (12)	872	1647	Lordsdale	New Orleans/279	novel	GII.3	4981	Sydney 2212	AY588132 (this study)
Sydney C14/02/AU (this study)	420	550	Hawaii	Mexico	novel	GII.3	5108	Bad Berleberg	AF409067
								Herzberg	AF539439
								Oberhausen 455	AF539440
								Paris Island	AY652979
								OS120458	AB071035
Picton/2003/AU (this study)	420	550	Pont de Roide AY682549	Richmond	novel	GII.1	5039	Gourdon 78	AY580335
Saitama U1/02/JP (10)	1527	1666	Lordsdale	Hawaii	GII.4	GII.12	5038	Honolulu	AF414420
								gifu 96	AB045603
								Schwerin	AF397905
								9912-02F	AB044366 (13)
Mc37/03/TH (11)	1527	1647	Lordsdale	New Orleans/306	GII.4	GII.10	5108	Vietnam 026	AF504671 (13)
								Vietnam 0703	AY237442 (13)
Snow Mountain 1/76/US (9)	420	1629	Hawaii	Melksham	novel	GII.2	4981	None found	NA
E3/1997/Crete (unpub.)	872	564	Lordsdale	Melksham	GII.4	GII.2	5068	None found	NA
VannesL23/1999/FR (unpub.)	815	576	MOH	Richmond	GII.5	GII.1/GII.12	5039	Tiffin	AY502010
S63/1999/FR (unpub.)	872	576	Melksham	MOH	GII.2	GII.5	5117	None found	
WUGI/02/JP AB081723 (10)	3370	1620	Southampton/91 L07418	BS5 AF093797	GI.4	GI.2	5359	None found	

*All strains belong to genogroup II except for WUGI/02/JP, which belongs to genogroup I. †RdRp, RNA-dependent RNA polymerase. ‡Strain used to determine breakpoint, closest matching strain in the database where enough sequence data were available for analysis. GenBank accession nos. are in Figure 1 unless stated. §For NoV GI (strain WUGI/02/JP), the classification system of Katayama et al. (10) was used; for GII (all other strains), the classification system of Vinjé et al. (23) was used. Closely related sequences are underlined. ¶Breakpoint determined by using the method of Smith (20) relative to Lordsdale nucleotide position for NoV GII (open reading frame [ORF]1/ORF2 overlap 5085–5104) and Norwalk for NoV GI (ORF1/ORF2 overlap 5358–5374). p value <0.0001.

The aims of this study were to characterize and compare 3 recombinant NoV sequences isolated in Sydney with other published recombinant NoV and those identified through database searches and phylogenetic analysis. The genetic relationship among all identified recombinants was explored and the recombination breakpoint accurately determined. A model of NoV recombination is proposed.

## Methods

Stool samples were thawed on ice from storage at –80°C and a 20% (vol/vol) stool suspension of total volume 1 mL made in water (pH 7.0). The sample was centrifuged for 1 min at 13,000 × *g*; the supernatant was then removed and centrifuged for a further 7 min at 13,000 × *g*. Viral RNA was extracted by using the QIAmp Viral RNA kit (Qiagen, Hilden, Germany) according to manufacturer's instructions. Amplification of the capsid region and a portion of the polymerase region was carried out as described previously (17). Amplification of a 507-bp region of the putative recombinant Sydney 2212/98/AU (corresponding to nucleotides 4610–5117 in Lordsdale virus, GenBank accession no. X86557) encompassing the 3´ end of the polymerase region and the 5´ end of ORF2 was achieved by using a nested reverse transcription–polymerase chain reaction approach. In brief, outer primers CB1 (17) and NoV2oR (5´-GTR AAC GCR TTY CCM GC-3´) (R = A or G, Y = C or T, M = A or C) and inner primer pairs 2212F (5´-GTG AGC ACA GAT ATM AAM TTA-3´) and 2212R (5´-AGA TGG AGY GGC GTC ATT CG-3´) were used in reaction conditions, as described previously (17). The ORF1/ORF2 overlap and flanking polymerase and capsid regions of another 2 suspected recombinants, NoV/Sydney C14/02/AU and NoV/Picton/03/AU, were amplified with hep170 (5´-TCH TTY TAT GGT GAT GA-3´) and GV29 (5´-CAA GAM ACW GTR AAM ACA TCA TCM CCA G-3´) (W = A or T) to produce a 1,070-bp product. Products were sequenced directly on an ABI 3730 DNA Analyzer (Applied Biosystems, Foster City, CA, USA).

Recombinant NoV were identified by constructing 2 phylogenetic trees, 1 using 420 bp of the 3´ end of the RNA polymerase region and the other using 550 bp of the 5´ end of ORF2. Strains that did not cluster with the same group of viruses in both trees were considered putative recombinant strains. Evolutionary distances between sequences were determined by using the GCG program, DNAdist (Kimura 2-parameter method) (18). The computed distances were used to construct phylogenetic trees with Fitch (18). To gain an internal estimate of how well the data supported the phylogenetic trees, bootstrap resampling (100 datasets) of the multisequence alignments was carried out with the program Seqboot (18). The consensus tree was calculated with Consense (18). Tree branch lengths were determined by analyzing the consensus tree with Puzzle, and trees were plotted by using the program TREEVIEW (version 1.6.6) (19).

The recombination breakpoint of putative recombinant strains was determined by using 2 methods: the maximum chi-squared method (20) and Simplot (version 2.5) (21). The maximum chi-squared method is recognized as being among the most accurate when compared independently with 13 other methods (22).

## Results

### Recombinant NoV Strains in Australia

#### Sydney Cluster Strain NoV/Sydney 2212/98/AU

In 1998, a number of outbreaks of gastroenteritis occurred within daycare centers across Sydney. The etiologic agents were identified as several closely related NoV GII strains, collectively termed Sydney cluster (17). We previously reported that the closest matching strain based on sequence searches using a 298-bp fragment of the RdRp region was the Arg320/95/AR strain (17), a known recombinant NoV (12) (Table). Further sequencing a Sydney cluster isolate, Sydney 2212 (NoV/Sydney 2212/98/AU, GenBank accession no. AY588132) was carried out to determine if this strain, like Arg320, was a recombinant NoV. The collated sequence data from this study and our previous study (17) were 2,446 bp long and encompassed 819 bp of the polymerase gene and the entire capsid region. Phylogenetic analysis of Sydney 2212 placed the polymerase region within the GII.4 cluster (based on the clustering system of Vinjé et al., 2004 [23]), which includes Lordsdale virus, but the capsid region grouped within the GII.3 cluster, which includes the prototype Mexico virus and New Orleans/279 (GenBank accession no. AF414412). Collectively these data demonstrate that Sydney 2212 is also a recombinant GII NoV.

#### NoV/Sydney C14/02/AU

**Figure 1:**
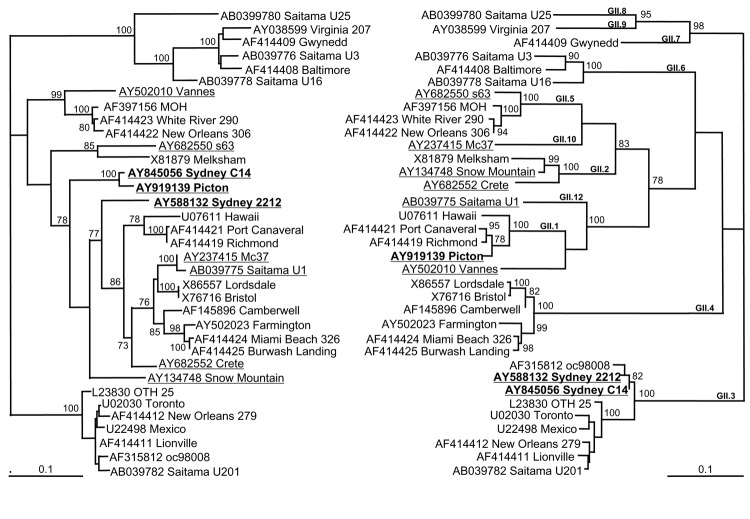
Phylogenetic analysis of the nucleotide sequences of capsid and polymerase regions of 9 identified recombinant norovirus genogroup II strains in relation to 26 known strains and prototype strains. The left tree analyzes the relationship of a 420-bp region of the 3´ end of the polymerase region. The right tree shows the relationship of 550 bp of the 5´ end of the capsid sequence. Suspected recombinants are underlined to emphasize their different phylogenetic groupings, and strains described in this study are represented in **boldface**. The percentage bootstrap values in which the major groupings were observed among 100 replicates are indicated. The branch lengths are proportional to the evolutionary distance between sequences and the distance scale, in nucleotide substitutions per position, is shown. The capsid clustering is shown in bold and is based on the classification of Vinjé et al. (23) (Table).

During February 2002, an outbreak of gastroenteritis occurred at a children's hospital in Sydney; it affected 21 children and staff. Phylogenetic analysis of the NoV strain (NoV/Sydney C14/02/AU, GenBank accession no. AY845056) responsible for the outbreak showed that the capsid clustered in the NoV GII.3 group, which includes prototype NoV strains Mexico and Toronto (Table, Figure 1). The polymerase clustered separately, but it was more closely related to the Melksham (GII.2) virus prototype than Mexico and Toronto viruses. The distinct segregation into 2 different phylogenetic positions strongly suggested that this virus was a recombinant NoV.

#### NoV/Picton/03/AU

In July 2003, an outbreak of vomiting and diarrhea affecting 71 patients and staff members occurred at an eldercare facility in New South Wales, Australia. The etiologic agent was a NoV GII strain designated NoV/Picton/03/AU (GenBank accession no. AY919139). Phylogenetic analysis of 550 bp of capsid sequence indicated that this strain clustered in the NoV GII.1 group, which includes the Hawaii prototype strain (Table, Figure 1). However, the polymerase did not cluster with Hawaii virus but with the second recombinant that we identified, namely NoV/Sydney C14/02/AU, and these isolates do not group with any known genotype in the polymerase region. Furthermore, although the capsid region demonstrated 94% sequence identity to Hawaii virus, the polymerase region was unrelated, showing only 85% nucleotide sequence identity. These results indicate that Picton/03/AU was also a recombinant NoV.

### Identification and Genetic Relationships

Systematic searches of the GenBank and EMBL databases and phylogenetic analysis identified a number of recombinants (Table). In GII, 9 independent recombinant NoV were identified. Two are published here, 4 were published previously (9–12), and 3 are unpublished. While 3 of the 9 recombinants were unique, 6 had 1–5 close relatives with >96% sequence identity in both polymerase and capsid regions. Sydney 2212/98/AU was closely related to the recombinant Arg320 in both polymerase and capsid regions (Table), with 97% and 96% nucleotide identity, respectively. The second recombinant we identified, Sydney C14/02/AU, was closely related to a number of strains of diverse geographic location, including Oberhausen 455/01/DE, 2 other German strains Bad Berleburg 477/01/DE and Herzberg 385/01/DE, the US isolate Paris Island/03/USA, and the Japanese isolate OS120458/01/JP (Table). The third recombinant NoV identified in the present study, Picton/03/AU, had 1 close relative identified by database searches, Gourdon78/00/FR. Four recombinants were identified that demonstrated >98% identity to the previously published recombinant, Saitama U1 (10), across both the polymerase and capsid regions: Honolulu314/94/US, Schwerin003/00/DE, Gifu'96/96/JP, and 9912-02F/99/JP. The Thai NoV recombinant Mc37/03/TH (11) had 2 close relatives from Vietnam, while the French recombinant Vannes L23/99/FR was closely related to an isolate from the United States, Tiffin/99/USA. Four additional NoV GII isolates have been reported as recombinants; however, we could not confirm these reports because the polymerase sequence data were not available. These isolates include Seacroft/90/UK (24), Wortley/1990/UK (24), Stepping Hill/01/UK (25), and Harrow/01/UK (25).

For NoV genogroup I, the aforementioned WUG1 (10) was identified as a recombinant (Table), and 2 other strains (NLV/BS5/98/DE, AF093797 and NoV/Saitama KU80GI/99/JP, AB058541) could not be ruled out as recombinants because their polymerase sequences did not cluster with any other strains. Thus, 24 recombinant NoV strains are known to exist: 3 new recombinants identified in the current study, 8 previously published (9–12), and 13 either newly identified or confirmed through database searches and phylogenetic analysis.

### Relationships between Regions of Recombinant NoV GII

To determine if genomic regions of the 9 representative recombinant NoV GII sequences (Table) were related to each other, phylogenetic (Figure 1) and pairwise sequence analyses (data not shown) were performed separately for the capsid region and the polymerase regions. Close relationships were found between sections of the identified recombinants (underlined in Table). The 2 Australian recombinants Sydney C14 and Picton were related to each other only in the polymerase region, with 96% nucleotide identity across a 420-bp fragment. However, their capsid regions were unrelated, showing only 73% nucleotide identity. In a similar fashion, the capsid region of Sydney 2212 was 98% identical to the capsid region of Sydney C14, while the polymerase region of Sydney 2212 shared only 85% identity with that of Sydney C14. The Japanese isolate Saitama U1 and the Thai strain Mc37 were related to each other only in the polymerase region, with 97% nucleotide identity across an 819-bp fragment, whereas alignments of their capsid regions demonstrated only 73% nucleotide identity.

### Recombination in the ORF1/ORF2 Overlap

**Figure 2:**
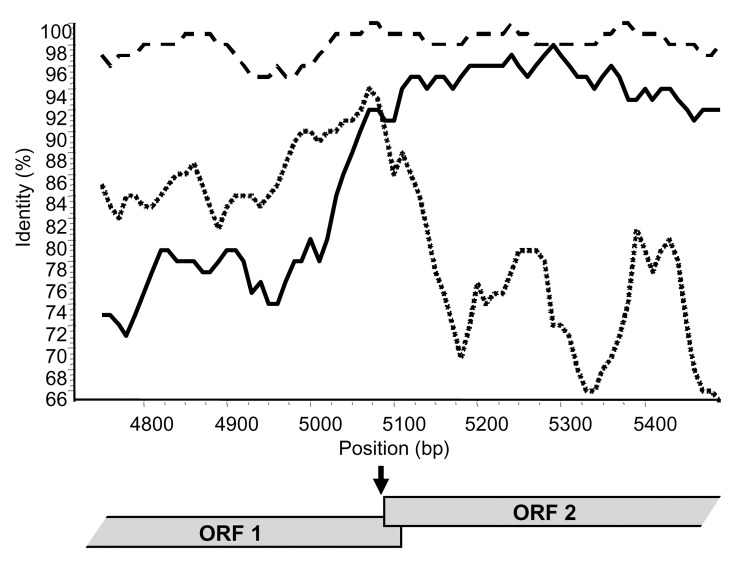
Similarity plot for Sydney 2212. The graph represents as a percentage the identity of the 2 putative parental strains, New Orleans/279 (black line) and Lordsdale (short dash), with the recombinant strain Sydney 2212. The window size was 100 bp with a step size of 10 bp. The site where the 2 parental strains have equal identity to the recombinant (i.e., where the lines cross) is the predicted site of recombination. By varying the window from 20 to 200 bp, the average recombination site was nucleotide 5081 with reference to Lordsdale. The percentage identity of Arg320 (long dash) to Sydney 2212 is also plotted. ORF, open reading frame.

By using the maximum chi-squared method (20), the recombination site was placed either immediately upstream (6/9 recombinants) or downstream (3/9 recombinants) of the 20-bp ORF1/ORF2 overlap in genogroup II strains (p<0.0003) (Table), and similar results were obtained by using Simplot (Figure 2). Recombination within the ORF1/ORF2 overlap cannot be specifically identified because this region is 100% conserved across all NoV GII sequences. Only 1 recombinant genogroup I strain has been identified, the Japanese isolate WUGI (10). The maximum chi-squared method placed the recombination point within the 17-bp ORF1/ORF2 overlap of this genogroup I isolate (p<0.0001) (Table).

## Discussion

We identified 3 recombinant NoV GII isolates responsible for outbreaks of acute gastroenteritis in New South Wales, Australia. Phylogenetic analysis of polymerase and capsid sequences of these and other recombinant NoV GII isolates showed 9 recombinant NoV GII sequences. All other recombinant NoV GII were close relatives of these (Table). The 3 NoV GII recombinant sequences identified in this study are constructed from only 2 polymerase sequences and 2 capsid sequences. They share either capsid or polymerase sequences, which suggests that the regions were derived from a pool of circulating viruses. The close geographic relationship of recombinants that share sequences in only 1 part of their genome may indicate the source location of the recombination event. In addition to the above example, this phenomenon is seen with 2 isolates from Vietnam, Vietnam 026 and Vietnam 0703, that share polymerase sequence with another Vietnamese isolate 9912-02F (Table) (13). However, the global distribution of recombinants such as Sydney C14, found in Australia, the United States, Germany, and Japan, is evidence against this hypothesis and indicates a much higher prevalence of recombinant strains in relation to other strains than was previously considered.

The putative crossover point was identified on either side of the overlap in 9 recombinant NoV GII (Table). Recombination within the overlap cannot be identified because it is 100% conserved across all NoV GII sequences and masks the breakpoint. This fact and the identification of the breakpoint at position 5359 in the recombinant NoV GI strongly suggest that recombination takes place within the reading frame overlap in NoV. The reading frame overlap and 6–7 bp of downstream sequence are closely related to sequence found at the start of the genome. In NoV GII are 28 bp that are highly conserved at both the 5´ end of ORF1 and around the ORF1/ORF2 overlap, with a consensus sequence of 5´-GTG AAT GAA GAT GGC GTC KAR YGA CGC Y-3´ (bases involved in the formation of stem loop structures are underlined). In NoV GI, 27 bp are highly conserved at the 5´ end of the genome and the ORF1/ORF2 overlap region with a consensus sequence of 5´-GYR AAT GAT GAT GGC GTC KAA RGA CGY-3´. The 2 highly conserved regions for NoV GI and GII contain 2 in-frame and 3 in-frame start codons, respectively. The duplication of a conserved sequence at the start of ORF1 and ORF2 is characteristic of caliciviruses and is seen in all 4 genera, NoV, sapovirus, lagovirus, and vesivirus (5,26–28). This repetition at the 5´ end of the 2 major ORFs led us to consider the role of ORF1/ORF2 as a negative-strand subgenomic RNA promoter site. Indeed, a subgenomic RNA promoter is required for subgenomic RNA synthesis and is often found in close proximity to the 5´ end of subgenomic RNA species (29). The presence of a subgenomic RNA has not been proven in NoV, but it is highly likely based on transcription in related viruses (26,27,30). For example, subgenomic RNA species have been identified, with 5´ ORF2 sequences, in 2 caliciviruses, namely, feline calicivirus (26,27) and rabbit hemorrhagic disease virus (RHDV) (30). The recent and first report of a calicivirus subgenomic RNA promoter in RHDV at the 5´ end of ORF2 provides evidence to support this hypothesis (30). Additionally, RNA promoter regions often have stem loop structures (reviewed in [31]); such structures have been identified within the repeated sequences found at the start of ORF1 and ORF2 of NoV (see sequences above) (28). Taken together, strong evidence exists that the conserved 27/28-bp sequence found at the 5´ end of the NoV genome and ORF2 is part of an RNA promoter sequence.

**Figure 3:**
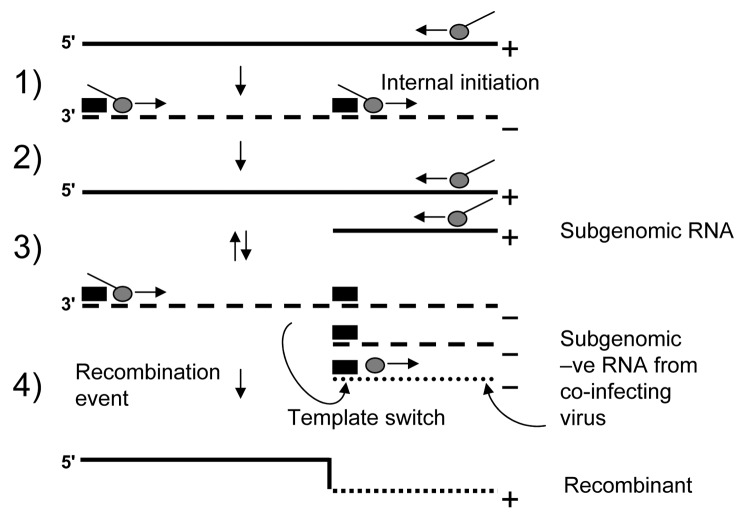
A simple mechanism for recombination in norovirus. 1) RNA transcription by the RNA-dependent RNA polymerase (RdRp) (gray circle) generates a negative-stranded intermediate (dashed line). 2) Binding of the RdRp to the almost identical RNA promoter sequences (filled boxes) generates positive-stranded (straight line) genomes and subgenomic RNA. 3) These templates direct RNA synthesis from the 3´ end that leads to the generation of both a full-length negative genome and a negative subgenomic RNA species. 4) Recombination occurs when the enzyme initiates positive-strand synthesis at the 3´ end of the full-length negative strand, stalls at the subgenomic promoter, and then template switches to an available negative subgenomic RNA species generated by a co-infecting virus. The net result is a recombinant virus that has acquired new open reading frame (ORF)2 and ORF3 sequences.

The primary mechanism involved in recombination in RNA viruses is the copy-choice model (32). In this model homologous recombination is driven by the viral encoded RdRp when pausing occurs during the transcription of a region of secondary structure. The polymerase then loses processivity and switches between RNA templates (reviewed in [7,8]). A number of models of subgenomic synthesis have been proposed, but the most widely recognized is the internal initiation mechanism (33). Here the replicase initiates positive-strand subgenomic transcription internally on a negative-strand copy of genomic RNA (29). Using these 2 well-supported models, we propose a simple mechanism for recombination in NoV (Figure 3). Replication and internal subgenomic RNA synthesis generate 2 positive RNA species. These templates direct RNA synthesis that leads to the generation of both a full-length negative genome and a negative subgenomic RNA species, in the second round of replication. The negative subgenomic RNA is the key player in our proposed model, and such species have been identified in viruses that produce subgenomic RNA (34). We propose that recombination occurs when the enzyme initiates positive-strand synthesis at the 3´ end of the full-length negative strand, loses processivity at the stem loop of the ORF1/ORF2 overlap, then hops across (template switching) to an available negative subgenomic RNA species generated by a co-infecting virus (Figure 3). Alternatively, the RdRp could also template switch directly from 1 genomic RNA to another genomic RNA in the highly conserved ORF1/ORF2 overlap. The net result of both possibilities is a recombinant virus that has acquired new ORF2 and ORF3 sequences.

The decline in the prevalence of previously dominant strains, such as US-95/96 in the United States and Australia (3,17), suggests immunity in the community might be an important factor in reducing further spread of NoV. Recombination offers NoV an attractive mechanism for immune evasion. Subgenomic RNA promoters have been proposed to be recombination hotspots (35,36). In this study we have presented data to support this hypothesis, and we have described a simple mechanism of how recombination might occur in NoV.
